# Measuring Center of Pressure Signals to Quantify Human Balance Using Multivariate Multiscale Entropy by Designing a Force Platform

**DOI:** 10.3390/s130810151

**Published:** 2013-08-08

**Authors:** Cheng-Wei Huang, Pei-Der Sue, Maysam F. Abbod, Bernard C. Jiang, Jiann-Shing Shieh

**Affiliations:** 1 Department of Mechanical Engineering, Yuan Ze University, Chung-Li 32003, Taiwan; E-Mails: s1005058@mail.yzu.edu.tw (C.-W.H.); s985051@mail.yzu.edu.tw (P.-D.S.); 2 School of Engineering and Design, Brunel University, London UB8 3PH, UK; E-Mail: maysam.abbod@brunel.ac.uk; 3 Department of Industrial Management, National Taiwan University of Science and Technology, Taipei 106, Taiwan; E-Mail: iebjiang777@gmail.com; 4 Center for Dynamical Biomarkers and Translational Medicine, National Central University, Chung-Li 32001, Taiwan

**Keywords:** center of pressure (COP), multivariate empirical mode decomposition (MEMD), multivariate multiscale entropy (MMSE)

## Abstract

To assess the improvement of human body balance, a low cost and portable measuring device of center of pressure (COP), known as center of pressure and complexity monitoring system (CPCMS), has been developed for data logging and analysis. In order to prove that the system can estimate the different magnitude of different sways in comparison with the commercial Advanced Mechanical Technology Incorporation (AMTI) system, four sway tests have been developed (*i.e.*, eyes open, eyes closed, eyes open with water pad, and eyes closed with water pad) to produce different sway displacements. Firstly, static and dynamic tests were conducted to investigate the feasibility of the system. Then, correlation tests of the CPCMS and AMTI systems have been compared with four sway tests. The results are within the acceptable range. Furthermore, multivariate empirical mode decomposition (MEMD) and enhanced multivariate multiscale entropy (MMSE) analysis methods have been used to analyze COP data reported by the CPCMS and compare it with the AMTI system. The improvements of the CPCMS are 35% to 70% (open eyes test) and 60% to 70% (eyes closed test) with and without water pad. The AMTI system has shown an improvement of 40% to 80% (open eyes test) and 65% to 75% (closed eyes test). The results indicate that the CPCMS system can achieve similar results to the commercial product so it can determine the balance.

## 1. Introduction

Postural stability is an important feature that protects people from falls and helps to complete the desired actions, which can be measured by the displacement of the center of pressure (COP) [1,2]. Due to the complicated and nonlinear information signals merging in the brain, the human brain reconstructs the environment from the incoming stream of often ambiguous sensory information and generates unambiguous interpretations of the world to control balance. There is a lot of related research about balance control [3], however, this study is based on measuring the human COP [4] signal. It is hoped to establish a balance measurement system to receive COP signals and to distinguish their characteristics, then find the COP signal difference of these multiple sources of sensory information between easy-fall elderly, normal elderly, and normal people. Due to the complicated and nonlinear nature of COP signals, the multivariate empirical mode decomposition (MEMD) would be a good candidate for decompose these signals. After filtering the COP signals, the multivariate multiscale entropy (MMSE) and complexity index (CI) need to be defined to evaluate how good it is for you to control your body in a stable condition. A single value named complexity index (CI) was obtained as the area under the MMSE curve. It gives us an index for assessing the degree of human body balance.

There are many commercial COP measurement systems [such as Advanced Mechanical Technology Incorporation (AMTI), Kistler, CATSYS 2000, *etc.* [5–7]]. These systems are a PC-based test systems, which communicate with a PC via a serial link, and data are recorded using different sensors. These force plates are specifically designed for use in gait and balance analysis. The systems are valuable for scientific researchers working on diseases, drug development, and monitoring of personnel. However, their prices are usually too high to be affordable for use in homecare systems. Hence, it is necessary to create a cheaper balance measurement system, with similar performance as commercial COP measurement systems, for preventing falls of elderly at home for the rapid growth of the aging population in the developing (e.g., China) and developed countries (e.g., USA, Europe, Japan). Furthermore, commercial systems can only collect the COP data and show the locus on screen, but do not provide analysis functions to assess the degree of human body balance. However, it is hard to see any characteristic patterns or indexes from these complicated and highly nonlinear COP data loci. Fortunately, several non-linear theories have been developed (MEMD, MMSE, CI) which can be used to analyze COP data for assessing the degree of human body balance [2,8–10]. Hence, the main purpose of this study is to design a cheap and portable device, which allows people to use it at home, which can collect and analyze data to assess the degree of human body balance. Therefore, the design procedure for the center of pressure and complexity monitoring system (CPCMS) force platform system is illustrated in the second part. In order to investigate the feasibility of the CPCMS, static, dynamic, and correlation tests show were performed in the third part. The fourth section introduces the analysis algorithms used in this study. The empirical mode decomposition (EMD), and MEMD combining with multiscale entropy (MSE) and MMSE using different intrinsic mode functions (IMFs) were compared. The aim is to identify which combination and which IMFs to used that will be accurate and can distinguish the different sways for COP signals. Experimental work is planned for four different sways (*i.e.*, eyes open, eyes closed, eyes open with water pad, and eyes closed with water pad) and compared with a commercial COP measurement system (AMTI). In parts five and six experimental results were analyzed and interpreted.

## 2. The Design of COP Measurement Device

**Figure 1.**
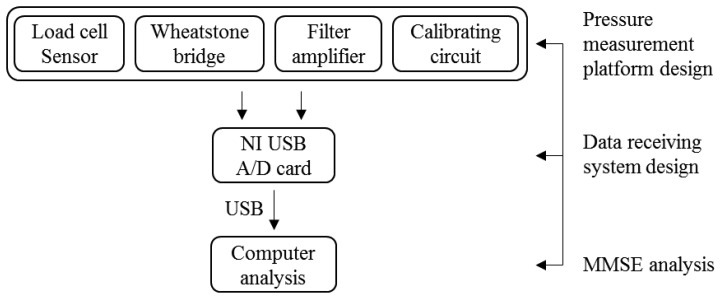
The center of pressure and complexity monitor system (CPCMS) design flow chart.

The structure of the balance measurement device is shown in Figure 1. This device consists of two systems, the pressure measurement platform and the data receiving systems. When the subject stands on the balance measurement device, the pressure measurement platform can receive raw COP signals. Then the data receiving system can convert the raw COP analog signals into digital signals for use in a computer or display device. A high-resolution data logging system is used with a 16-bit A/D card (*i.e.*, National Instruments (NI) USB-6212) to provide accurate measurement data. The system is named Center of Pressure and Complexity Monitor System (CPCMS).

### 2.1. The Pressure Measurement Platform

The pressure measurement platform is designed to receive raw balance signal data. Because the load cell signals are very small and have added noise, the signals need to be amplified and filtered. Therefore, the pressure measurement platform system includes five parts; load cell sensor, Wheatstone bridge, filter circuit, amplifier circuit, and calibrating circuit. The pressure sensing principle of the electronic weight scale is the same as the strain gauge. When the electronic weight scale is under pressure, load cells in the scale will produce deformations. The resistance will change because of the deformation, but the resistance change is difficult to measure, so measurements were based on voltage changes rather than resistance changes. This principle is used as the basis for the measurement of body balance signals. The load cells are embedded in the four corners of the scale. Each load has three signal lines which use a Wheatstone bridge circuit to collect the output voltage changes due to pressure.

### 2.2. Data Receiving System Design

**Figure 2.**
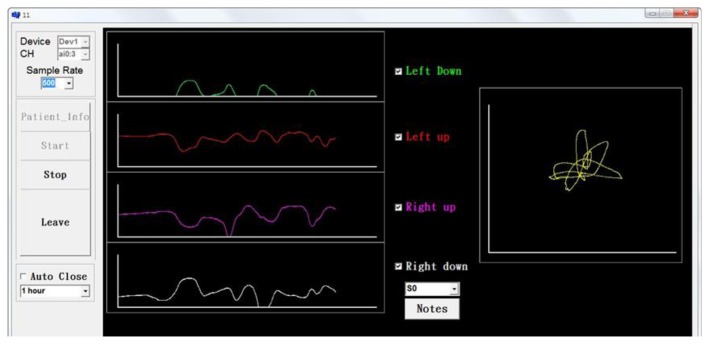
Data receiving program.

The data logging system uses 16 bits analog to digital (A/D) card to receive the voltage from the pressure measurement platform. After the A/D card converts the voltage signal into a digital signal, the data is saved then analyzed using Matlab. The NI USB-6212 A/D card samples the data at 400 kHz rate, and 16-bit resolution. Four analog input channels were used to receive the signals from the pressure measurement platform, and send the digital signals to a computer through high-speed USB data streams. C programming was used to develop the system which displays and saves the raw data (Figure 2).

### 2.3. Multivariate Multiscale Entropy Analysis

Multivariate Multiscale Entropy (MMSE) [8] analysis is a method of measuring the complexity of finite length time series. This computational tool can be applied both to physical and physiological data sets, and can be used with a variety of measures of entropy. More details about this analysis method are shown in Section 4.

## 3. CPCMS Test

### 3.1. Static Tests

**Figure 3.**
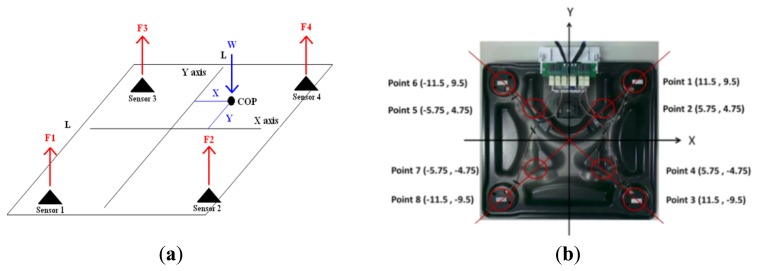
(**a**) The schematic diagram for calculating the COP location. (**b**) The schematic diagram of eight test locations.

The COP location is identified from the voltage signals, which are received from the four load cells. The schematic diagram of calculating the COP location is shown in Figure 3(a). Four load cells generate a reaction force when under pressure, and the reaction forces are F1, F2, F3, and F4. The distance between the sensors is L. W = F1 + F2 + F3 + F4, and COP = (X, Y). The formulas for calculating the COP location are as follows:

(1) $$X=\frac{[\left(F4+F2\right)-\left(F1+F3)\right]\times L}{W}$$

(2) $$Y=\frac{[\left(F3+F4\right)-\left(F1+F2)\right]\times L}{W}$$

In order to evaluate the static reproducibility, a bowling ball is placed at eight different locations, as shown in Figure 3(b). The CPCMS was calibrated using static state tests. Each location was tested 10 times and the COP location is drawn on a figure. The mean and standard deviation values of each location were also calculated. The results shown in Table 1 indicate the mean of the error ratio for X direction is 7.5% so the maximum error distance for X direction is 0.86 cm (the farthest X coordinate 11.5 cm multiply mean of the error ratio 7.5%), and the mean of the error ratio for Y direction is 11.5% so the maximum error distance for Y direction is 1 cm (the farthest Y coordinate 9.5 cm multiply mean of the error ratio 11.5%).

For the distance from the origin the mean of the error ratio is 9%, so the maximum error distance for distance from origin is 1.3 cm (the longest distance from origin 14.91 cm multiplied by the mean of the error ratio 9%). The error ratios for the X direction, Y direction, and distance from origin are 7.5%, 11.5% and 9%, respectively. In applied practice, confidence intervals are typically stated at the 95% confidence level [11]. Practically, 10% error gives a 95% confidence level. Therefore, the error for every time COP is measured using the CPCMS is around 10% so the static tests for CPCMS can be accepted.

### 3.2. Dynamic Tests

**Figure 4.**
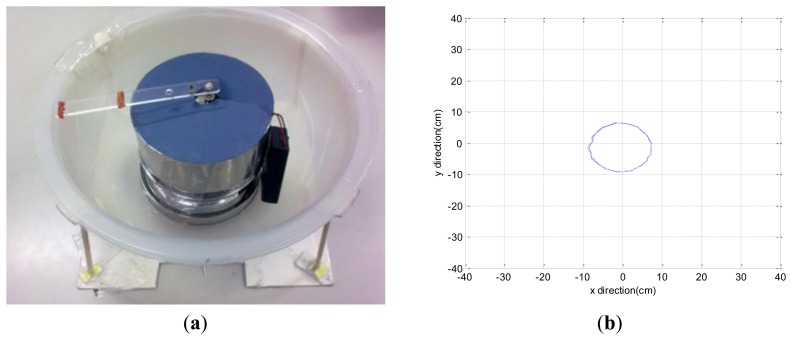
(**a**) The dynamic simulating device. (**b**) The regular displacement figure measured by CPCMS.

In order to evaluate the reproducibility of the dynamic state displacement, a dynamic simulating device is designed Figure 4a in order to generate regular dynamic displacement. Driven by high torque motors, a weighed ball rolls on the surface. Therefore, a regular circle of displacement is generated as shown in Figure 4b, and the circle is plotted using radius as the mean of total excursion plus the standard deviation of all excursions (mean + SD) [12]. The mean and standard deviation values of the radius after ten times testing were calculated. The results are shown in Table 2. The real radius for simulating device is 10 cm, and the error ratio for totally 10 times tests is 4% equal to 0.4 cm. The dynamic test error ratio 4% is better than the static test error ratio of 10% for CPCMS.

### 3.3. Correlation Analysis Test

**Figure 5.**
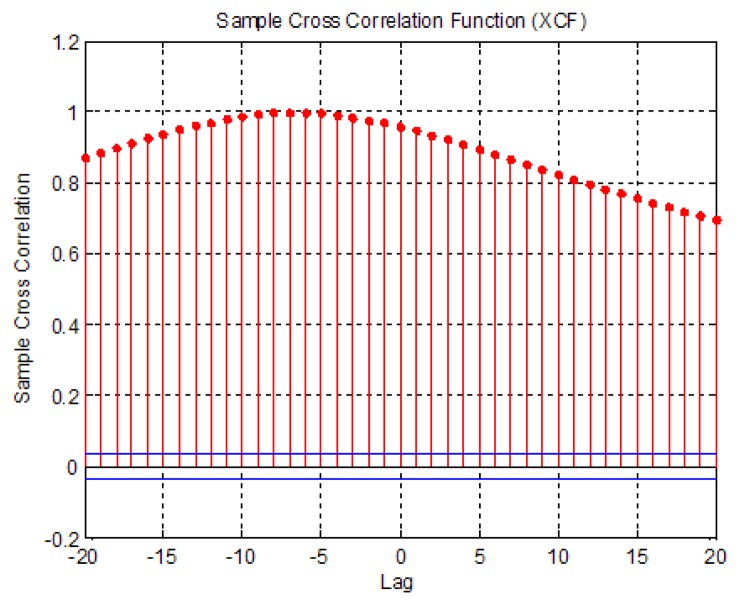
Two data are similar.

The correlation of the two different signals can be determined using a cross-correlation function. If two signals are similar to each other, the correlation value will be close to 1. Conversely, if two signals are not similar, the correlation value will go to −1 (Figure 5). The maximum correlation values were also identified. The raw COP data measured with the AMTI and CPCMS were aligned to the same start point before calculating the correlation value.

The correlation results are shown in Table 3. It is found that the correlation values are close to 1. The correlation value of sway 4 type is the smallest one of the four sways, but still close to 1. It means that the COP sway data measured by the two systems are similar.

### 3.4. Intraclass Correlation Coefficient Test

Intraclass correlation coefficient (ICC) has been frequently used for reliability of measurements [13]. ICC values of bigger than 0.75 represent “excellent reliability” and values between 0.4 and 0.75 represent “fair to good reliability” [14]. Static and dynamic tests data were used to calculate the ICC. In static tests the ICC for top right sensor X is 0.66 and Y is 0.99, bottom right sensor X is 0.97 and Y is 0.99, top left sensor X is 0.87 and Y is 0.92, and bottom left sensor X is 0.74 and Y is 0.98. In the dynamic test the ICC is 0.88. From the results it can be seen that the CPCMS has excellent reliability except for the top right sensor X = 0.66) and bottom left sensor (X = 0.74).

## 4. Analysis Algorithms

The COP measurement data were analyzed using empirical mode decomposition (EMD) and MEMD for detrending the data. Since the data is contaminated with noise, it needs to be filtered prior to analysis. The EMD method is an iterative signal processing algorithm which decomposes the intrinsic components from signals by iterative sifting processes. Then, MSE is used to evaluate the body COP. MEMD is a newly modified method originally from EMD, primarily is to create white noise, of the length as the original signal, and then feed the original signal and white noise in different channels. MEMD is not only suitable for dealing with multichannel signals, but also solve the problem of mode mixing addition of white Gaussian noise to different channels (N-A MEMD) [9].

MSE analysis is a new method of measuring the complexity of finite length time series. Entropy-based algorithms for measuring the complexity of physiological time series have been widely used. They have proved to be useful in discriminating between healthy and disease slates, although some results may generate misleading conclusions [10]. Recently proposed latest multivariate multiscale entropy (MMSE) [15] analysis method can compute both channels of data. MMSE consolidates the difference of M-L and A-P directions that can be seen the improvement in the overall balance, rather than simply just looking at the M-L or A-P direction. The degree of improvement can be seen more obviously.

### 4.1. Empirical Mode Decomposition

The EMD method is a signal processing and analysis theory applied to nonlinear or non-steady-state system signals. Its function is separated from the non-linear and non-steady-state signal representing different actuation mechanism of the reaction. The EMD has groups of intrinsic mode functions (IMF) that correspond to the modal function within the system with different mechanisms of reaction. The EMD method is based on the signal in the local maximum and local minimum value to define the upper and lower envelope. The upper envelope is the cubic spline to link with the local maximum value of the continuous curve, and the lower envelope is the cubic spline to connect to the local minimum value of the continuous curve. Average upper and lower envelope are seen as a signal of a trend. The average envelope can be regarded as a possible intrinsic mode functions.

Separating the possible intrinsic mode functions from the signal process is called the sifting process. The intrinsic mode functions obtained from the sifting process must satisfy the following two conditions, otherwise we must run the sifting process again [16]:

(1) In the whole time series, the number of all local extrema and zero-crossing difference cannot be more than one.
(2) In any one time point, the average envelope must tend to zero.

If these two conditions are satisfied, the separated signals called intrinsic mode functions (IMF) will be recorded as *c_1_*. Original signal and *c_1_* subtraction can get the residual signal. The residual signal can be input as the decomposition of next intrinsic mode functions. Repeating this process can be gradually decomposed for different intrinsic mode functions until the residual signal is a monotonic function.

The original signal after the n times decomposition can get different n groups of IMF signals. The relationship between signals decomposed by the empirical mode decomposition method and the original signal can be expressed by the following Equation (3):

(3) $$X\left(t\right)=\sum_{i=1}^{n}c_{i}+r_{n}$$

where *X (t)* is original signal, *c_i_* represents the number *i* IMF, *r_n_* is number n times residual signal.

### 4.2. Multivariate Empirical Mode Decomposition

EMD is an algorithm proposed by Huang *et al.* [17]. It has been widely used in nonlinear and non-stationary data analysis based on the inherent characteristics of the time series. In order to diversely extend the application of EMD, the MEMD method was being developed to decompose diversely nonlinear and non-stationary signals [18]. The MEMD method not only overcomes the limit of only a single input in the EMD method, but also addresses the fact that noise signal residues in different channels after adding white noise will be caused in the signal processing required to spend in lengthy problems. In addition, it is similar to the empirical mode decomposition as a dyadic filter in a multivariable input of each channel. It has an advantage on correction in aligning the corresponding IMFs from the different channels to the same frequency range [9].

In the multivariate empirical mode decomposition method, the average value of m (t) is calculated by multivariate enveloped K direction vectors, such as shown in Equation (4) below:

(4) $$m\left(t\right)=K^{-1}\sum_{k=1}^{K}e^{\theta_{k}}\left(t\right)$$

In which the ${\left\{e^{\theta_{k}}\left(t\right)\right\}}_{k=1}^{K}$ is a multivariable envelope in the vectors, along the K direction that predict multi-channel input s (t) and multivariate IMF calculations via the s (t)-m (t) and stopping criteria. This process is repeated until all predicted standard signals are satisfied and the standard empirical mode decomposition stops.

### 4.3. IMF Selection

In order to obtain the signal after filtering, several IMFs both in EMD and EEMD [19] have to be combined. Choosing the right IMFs from the original signal is very important and challenging. For example, considering brain signals, the frequency for brain signals is 0.5 Hz to 30 Hz for δ, θ, α, and β signals. Taking the Fourier transform for each IMF can indicate the frequency range of the original signal. According to the brain signal frequency, IMFs that satisfy 0.5 Hz to 30 Hz are usually selected. Finally, combining the IMFs, the original brain signal can be reconstructed without the noise [20]. The same methodology is used to filter the COP signal. Selecting the frequency range is usually done based on trial and error, however, recently a study [21] has reported that the human body center of pressure signal is lower than 2 Hz. So, a 2 Hz reference was chosen for analysing the IMFs.

### 4.4. Multiscale Entropy

Entropy-based algorithms have been widely used for measuring the complexity of physiological time series. They have proved to be useful in discriminating between healthy and diseased patients [22], although some results may generate misleading conclusions. For example, the entropy that these algorithms assign to time series derived from the ventricular response in atrial fibrillation (AF) is higher than that is assigned to sinus rhythm time series derived from healthy subjects [23]. However, healthy systems generate much more complex outputs than diseased ones. Traditional algorithms are single-scale based and fail to account for the multiple time scales inherent in physiological systems.

The multiscale entropy (MSE) developed by Costa *et al.* [10] is used to evaluate the complexity of signals over different time scales. Based on the approximate entropy, this method uses Sample Entropy to quantify the regularity of the finite length time series, which has been applied effectively in analysis of physiology, biology, and geosciences data [23–25].

Given a one-dimensional discrete time series {*x_i_*… *x_n_*}, MSE first constructs multiple coarse-grained time series by the scale factor *τ*. Each element of the time series, {*y*^(1)^}, is according to Equation (5):

(5) $$y_{j}^{\left(\tau \right)}=1/\tau \sum_{i=\left(j-1\right)\tau +1}^{j\tau }x_{i}\;1\leq j\leq N/\tau$$

For scale one, the time series {*y*^(1)^} is simply the original time series. The length of each coarse-grained time series is equal to the length of the original time series divided by the scale factor *τ* .Then, we can calculate the Sample Entropy for each coarse-grained time series plotted as a function of the scale factor *τ* [10]. Sample Entropy reflects the conditional probability that two sequences of m consecutive data points which are similar to each other will remain similar when one more consecutive point is included, and being “similar” means that the value of a specific measure of distance is less than r [9]. The complexity degree of different combinations in each direction is measured in terms of the complexity index (CI), which is defined as the area under the MSE curve over all scales, $CI=\sum_{i=1}^{\text{scale}}\text{SampEn}\left(i\right)$, in which scale is the maximum of the scale factors [2,26]. It gives us an index for assessing the degree of human body balance.

### 4.5. Multivariate Multiscale Entropy

MMSE [27] calculates the relative complexity of the multichannel signals through the plot of the multivariate sample entropy. This makes it possible to assess the structural complexity of multivariate physical or physiological systems, together with more degrees of freedom and enhanced rigor in the analysis. In the MMSE, if the multivariable sample entropy value is higher than most of the other scales, multivariable time-series are considered to be more complex than the others. This is the same with the original MSE. Applications include the analysis of pathological heartbeat conditions, electroencephalograms (EEG), postural sway dynamics analysis, and the complex dynamics of human red blood cells [28].

## 5. Experimental Design

In order to prove that CPCMS can estimate the different sizes of the different sways, four sway tests types were selected to produce different sway displacement. The sampling rates of the two systems are both 50 Hz, measured for 60 s. The subjects were 20 young adults, aged between 20–25 years old. The four steps of the experimental are as follows:

Test 1. The subject with eyes open standing on the balance measurement system and measured for one minute (EO).
Test 2. The subject with eyes closed standing on the balance measurement system and measured for one minute (EC).
Test 3. The subject with eyes open standing on the water pad placed on the balance measurement system and measured for one minute (WPEO).
Test 4. The subject with eyes closed standing on the water pad placed on the balance measurement system and measured for one minute (WPEC).

The AMTI system has an offset function so our system and water pad will not affect the AMTI system and can record COP data at the same time. Signals were compared for both eyes open and both eyes closed with and without water pad (EO and WPEO, EC and WPEC). The CI value for WPEO and WPEC should be lower than EO and EC. It is expected that the CPCMC system can get the same result as the AMTI system.

## 6. Results Analysis

### 6.1. Find COP Signal Frequency and Choose IMF

The COP displacements in two directions of anterior-posterior (AP) and the medial-lateral (ML) are often used to characterize the COP stabilogram [29]. Before performing the EMD and MEMD analysis, the IMF selection has to be made. MEMD was performed first, then the Fourier transform for each IMF to get the frequency range. In this experiment, 20 subjects were tested using both the AMTI and the CPCMS systems. The frequencies for each IMF are shown in Table 4 and Table 5 for the CPCMS and the AMTI respectively. IMF 5+6 were selected since these indicate less than 2 Hz signals.

### 6.2. Comparing EMD-Enhanced MSE Using IMF 2+3 and IMF 5+6

In a previous study by the authors [30], EMD-Enhanced MSE was used to analyse the data using IMF 2+3 (selected using trial-and-error). For this study, the comparison is shown in Table 6. From Table 6, CPCMS results (eyes open case) does not show any improvement, but for the eyes closed case, improvement range between 40% to 50%. AMTI (eyes open case) has an improvement between 25% to 35%. Finally, for the eyes closed case an improvement between 40% to 65% is reported. The p-valued in the AP direction both are less than 0.05. According these results, it can see that IMF 5+6 is better than IMF 2+3, so in the following analysis IMF 5+6 will be used.

### 6.3. Comparing EMD-Enhanced MSE and MEMD-Enhanced MSE Using IMF 5+6

EMD-Enhanced MSE and MEMD-Enhanced MSE using IMF 5+6 the results comparison is shown in Table 7, which show an improvement of CPCMS for the eyes open case between 30% to 35% and for the eyes closed case between 50% to 60%. While the AMTI method for eyes open case has an improvement between 35% to 40%, it has no improvement for the eyes closed case. For the AP direction both the p-values are less than 0.05. This comparison shows that using MEMD is better than EMD, so MEMD with IMF5+6 is used as the main analysis method.

### 6.4. Comparing MEMD-Enhanced MSE and MEMD-Enhanced MMSE Using IMF 5+6

MEMD-Enhanced MSE and MEMD-Enhanced MMSE using IMF 5+6 the comparison is shown in Table 8. From Table 8, it can be seen that there is an improvement in CPCMS for eyes open from 35% to 70% and for eyes closed from 60% to 70%. For AMTI has an improvement for eyes open from 40% to 80%, for eyes closed from 65% to 75%. Both p-values of ML and AP directions are less than 0.05. In this comparison, it has been improved significantly, so we think MEMD-Enhanced MMSE is better than MEMD-Enhanced MSE.

## 7. Discussion and Conclusions

For data analysis algorithms, it is expected that the CI value for WPEO and WPEC should be lower than EO and EC because when subjects stand on a water pad they need to spend more effort to keep their balance for prevent falling than when standing on the platform directly. This behavior will cause the complexity index to reduce. For the eyes open case, since there is visual assistance, it is easier to keep their balance than with eyes closed. Therefore, it is more difficult to distinguish EO and WPEO of these two sways. But, when using MEMD-enhanced MMSE, there are more subjects to be distinguished. Furthermore, when comparing EMD-enhanced MSE, MEMD-enhanced MSE, and MEMD-enhanced MMSE, distinguishing the different sways is clearer. However, the reason behind this is that in our experiment subjects are young and healthy. The human body balance is based on powerful perception source of variety of sensory information, including the combination and integration of visual, tactile, and auditory inputs [31]. For young and healthy people their adaptability is better due to their perception sources being better than elderly people, so they can remain balanced with eyes closed or standing on a water pad easily. However, age and illness are thought to cause partially loss of the ability to judge body placement. Hence, if elderly people were tested, because their adaptabilities are perhaps not as good as those of young and healthy people, they will lose balance earlier with eyes closed or standing on a water pad. Obviously, due to the complicated and nonlinear information signals merging in the brain, the human brain reconstructs the environment from the incoming stream of often ambiguous sensory information and generates unambiguous interpretations of the world to control the sense of balance [3,32]. Therefore, via the cheap and portable CPCMS system that has been developed in this study, it is worth to investigate in the next step how these multiple sources of sensory information will affect this merging of the sensory information in the brain in terms of the different combinations and integration strategies when degradable sensor information has occurred due to aging and diseases.

For IMF selection, using IMF 5+6 gives better results, as confirmed by Freitas *et al.* [21] for COP signals below 2 Hz. Therefore, IMF 5+6 is used for all the analysis procedures in this study for young and healthy people. However, for elderly people, the IMFs may be in different ranges. This needs to be experimented to verify the analysis results for elderly people. Hence, the merit of the extended empirical mode decomposition is to decompose the complicated signals to several IMFs which allows us to pinpoint what kind of physiological event will cause these IMFs. This method has created a good approach for investigating how all these different sources of information have to be efficiently merged to form a coherent and robust perception in the brain [33] and how our brains control muscles for keeping balance from different sensors.

The cost of the developed CPCMS is around 1,300 USD, but for AMTI it is around 25,000 USD. The cost of the CPCMS is very low compared to AMTI. The sampling rate of both the CPCMS and AMTI can be adjusted by data acquisition program between 100 Hz and 500 Hz. Because the COP signal frequency is very low (less 2 Hz), CPCMS is therefore set to 100 Hz in the experiment. The analog inputs for CPCMS and AMTI are 16-bit. The dimension of CPCMS is 31 × 31 × 4.7 cm, while the AMTI is 46.3 × 50.8 × 82.6 cm (W × L × H). The weight for CPCMS and AMTI are 2 kg and 28 kg, respectively, so CPCMS is very light and portable. In the next step, we will use a new 16 bit AD chip which is cheaper than the NI A/D card (cost around 20 USD). Hopefully, it will bring the whole CPCMS cost down. Currently, only the static COP signal is measured when people are standing but it is worth to investigate the dynamic COP signals when people are walking which is more related to some neuroscience illness, such as Parkinson's and Alzheimer's diseases [34,35]. Therefore, pressure sensors will be inserted into the insole in order to measure dynamic COP signals when people are walking [36] in the near future.

From the analysis results in this study, CPCMS has similar results compared to the AMTI system. To conclude, a low cost and portable system (*i.e.*, CPCMS) for measuring and analysis COP signal has been designed which allows one to quantify the dynamic properties of humans' balance in order to assess the risk of falling among elderly people when dealing with a large population size via this more handy and convenient device in the near future. CPCMS can be also used in hospitals for doctors to assess the degree of dizziness and surgery recovery patients [30].

## Figures and Tables

**Table 1.** The mean and standard deviation values of eight locations (cm) and the distance to the origin measured by CPCMS.

**Verification Points**		**Average of measuring Points**		**Error Ratio**		**Distance from Origin (cm)**		

X (cm)	Y (cm)	X (cm)	Y (cm)	X (cm)	Y (cm)	Verification	Measuring	Error Ratio

11.5	9.5	11.2 ± 0.14	11.6 ± 0.15	2.6%	22%	14.91	16.1 ± 0.21	8%
5.75	4.75	6.2 ± 0.45	5.7 ± 0.42	8%	2%	7.45	8.4 ± 0.62	12%
11.5	−9.5	12 ± 0.13	−11 ± 0.12	4%	15%	14.91	15.7 ± 0.03	5%
5.75	−4.75	6.4 ± 0.15	−5.3 ± 0.16	11%	11%	7.45	8.3 ± 0.05	11%
−5.75	4.75	−6.9 ± 0.07	5.6 ± 0.05	2%	17%	7.45	8.8 ± 0.09	18%
−11.5	9.5	−11.2 ± 0.27	10.6 ± 0.2	2%	12%	14.91	14.9 ± 0.34	0%
−5.75	−4.75	−7.3 ± 0.18	−4.8 ± 10.17	27%	1%	7.45	8.7 ± 0.25	16%
−11.5	−9.5	−11.2 ± 0.23	−10.6 ± 0.2	3%	12%	14.91	15.4 ± 0.3	3%

			Mean	7.5%	11.5%		Mean	9%

**Table 2.** The mean and standard deviation values of the radius measured by CPCMS

**Times**	**1**	**2**	**3**	**4**	**5**	**6**	**7**	**8**	**9**	**10**	**Mean ± SD**
Real Radius (cm)	10	10	10	10	10	10	10	10	10	10	10 ± 0
Measure Radius (cm)	9.2	9.18	9.8	9.73	9.55	9.66	9.33	10.01	10.19	10.02	9.66 ± 0.35
Error Ratio	8%	8%	2%	3%	4%	3%	7%	0%	2%	0%	4 ± 3%

**Table 3.** The four sway signal correlation results (cross-correlation).

	**X**	**Y**
Sway 1	0.987 ± 0.014	0.988 ± 0.017
Sway 2	0.989 ± 0.014	0.993 ± 0.005
Sway 3	0.986 ± 0.019	0.991 ± 0.01
Sway 4	0.981 ± 0.024	0.983 ± 0.02
Mean±SD	0.986 ± 0.018	0.989 ± 0.013

**Table 4.** IMF frequencies for CPCMS.

**CPCMS** Frequency (Hz)	**Mean ± SD**	
	X	Y
IMF 2	9.17 ± 1.74	9.53 ± 1.59
IMF 3	4.22 ± 0.84	4.54 ± 1.04
IMF 5	1.24 ± 0.26	1.22 ± 0.24
IMF 6	0.73 ± 0.18	0.73 ± 0.15

**Table 5.** IMF frequencies for AMTI.

**AMTI** Frequency (Hz)	**Mean ± SD**	
X	Y
IMF 2	8.59 ± 1.05	7.97 ± 0.75
IMF 3	4.59 ± 0.56	4.84 ± 0.59
IMF 5	1.3 ± 0.19	1.27 ± 0.16
IMF 6	0.76 ± 0.13	0.77 ± 0.11

**Table 6.** Compare EMD-Enhanced MSE use IMF 2+3 and IMF 5+6 (CI-EO&WPEO is to compare complexity index for eyes open and water pad with eyes open, CI-EC&WPEC is to compare complexity index for eyes closed and water pad with eyes closed).

**CPCMS**	**CI-EO&WPEO**		**CI-EC&WPEC**	

	ML	AP	ML	AP

IMF 2+3 p-value IMF 5+6 p-value	30%		40%	

	0.72	0.009	0.559	0.006

	30%		50%	

	1	0.04	0.12	0.028

**AMTI**				

IMF 2+3 p-value IMF 5+6 p-value	25%		40%	

	0.55	0.124	0.615	0.059

	35%		65%	

	0.032	0.028	0.001	0.041


**Table 7.** Compare EMD-Enhanced MSE and MEMD-Enhanced MSE use IMF 5+6.

**CPCMS**	**CI-EO&WPEO**		**CI-EC&WPEC**	

	ML	AP	ML	AP

EMD IMF 5+6 p-value MEMD IMF 5+6 p-value	30%		50%	

	1	0.04	0.12	0.028

	35%		60%	

	0.429	0.022	0.64	0.045

**AMTI**				
	
EMD IMF 5+6 p-value MEMD IMF 5+6 p-value	35%		65%	

	0.032	0.028	0.001	0.041

	40%		65%	

	0.6	0.002	0.245	0.014


**Table 8.** Compare MEMD-Enhanced MSE and MEMD-Enhanced MMSE use IMF 5+6.

**CPCMS**	**CI-EO&WPEO**		**CI-EC&WPEC**	

	ML	AP	ML	AP

MEMD MSE IMF 5+6 p-value	35%		60%	

	0.429	0.022	0.64	0.045

		ML&AP		ML&AP	

MEMD MMSE IMF 5+6 p-value	70%		70%	

	0.044		0.048	

**AMTI**	ML	AP	ML	AP

MEMD MSE IMF 5+6 p-value	40%		65%	

	0.6	0.002	0.245	0.014

		ML&AP		ML&AP	

MEMD MMSE IMF 5+6 p-value	80%		75%	

	0.037		0.048	

